# Signatures of the maternal-infant oral microbiome in contexts of imprisonment and social vulnerability

**DOI:** 10.3389/fdmed.2026.1805944

**Published:** 2026-06-11

**Authors:** Ninfa Ramírez-Durán, Gauddy Lizeth Manzanares-Leal

**Affiliations:** Laboratorio de Investigación en Microbiología Médica y Ambiental, Facultad de Medicina, Universidad Autónoma del Estado de México, Toluca, Estado de México, México

**Keywords:** 16S rRNA gene amplicon sequencing, maternal and child health, oral microbiome, social determinants of health, vulnerable populations

## Abstract

**Introduction:**

The oral microbiome is a dynamic ecosystem that develops in early life through physiological processes and environmental exposures and plays a key role in oral and systemic health. In socially vulnerable settings, such as prisons, altered living conditions may shape the establishment of the pediatric oral microbiome. This study aimed to characterize the oral microbiome signatures in mothers, children, and pregnant women living in prisons, and to explore microbial variation in relation to confinement-related living conditions.

**Methods:**

A cross-sectional study was conducted that included the entire maternal-child population (*n* = 43 samples) residing in five prisons and social reintegration centers in the State of Mexico. Saliva and oral biofilm samples were collected from mothers (*n* = 19), children (*n* = 19), and pregnant women (*n* = 5). The taxonomic composition was evaluated by sequencing the 16S rRNA gene. Analyses of alpha diversity (Shannon index), beta diversity (weighted UniFrac), and differential abundance (DESeq2) were performed.

**Results:**

A shared core microbiome, dominated by *Streptococcus* and *Veillonella*, was identified in all groups. However, significant differences in microbial diversity were observed according to prison (*p* = 0.03) and population group (*p* = 0.01). Children exhibited the lowest diversity and a pronounced dominance of *Streptococcus* (58%), consistent with microbiome profiles in the early stages of life but consistent with early-life microbiome profiles. Mothers showed a greater abundance of opportunistic environmental taxa, including *Pseudomonas* (13.2%) and *Raoultella* (5.2%). Pregnant women showed the greatest diversity and a distinctive signature characterized by enrichment of *Actinomyces* and *Leptotrichia*. Beta diversity analyses revealed partial overlap between mothers and children, suggesting shared microbial patterns potentially related to common living conditions.

**Conclusion:**

These findings provide an exploratory characterization of the oral microbiome in mothers, children, and pregnant women living in prison settings. The lower diversity observed in children, along with the presence of opportunistic environmental taxa, underscores the importance of accounting for confinement-related living conditions in oral health research and prevention strategies for historically marginalized populations. Future comparative and longitudinal studies are needed to clarify the relationship between prison-specific conditions and variation in the oral microbiome.

## 1 Introduction

People deprived of their liberty constitute a highly vulnerable population from a health perspective. Conditions of confinement, such as overcrowding, limited access to medical services, poor hygiene, and restricted food intake, create an environment that significantly impacts individuals’ health (1, 2). Close physical proximity in confined spaces such as prisons has been reported to be associated with an increased risk of transmission of infectious and communicable diseases (3). Furthermore, it has been suggested that people in prison experience psychological stress and a high burden of stressful conditions, leading to alterations in immune and cardiovascular functions (3). Despite general agreement that the prison environment is problematic, its effects on health are often expressed in general terms, with little quantitative data on health outcomes.

In Mexico, 86% of women in prison are mothers, 74% are between 18 and 44 years of age, the prime childbearing years, and two-thirds are the primary caregivers for children under 18 (4). In particular, women, children, and pregnant women living in prisons represent a doubly vulnerable group due to their specific biological needs and the absence of adequate health policies in these environments (1). Research suggests that people who give birth in prisons are at greater risk of receiving inadequate perinatal care and suffering prenatal and obstetric complications, such as low birth weight, compared to non-incarcerated populations (5).

Traditionally, the study of prison health has focused on infectious diseases such as tuberculosis, HIV, and sexually transmitted infections (6–8). However, it is increasingly important to understand how conditions of confinement are reflected in underexplored dimensions, such as the human microbiome. In particular, the oral microbiome, composed of bacterial communities that play a key role in oral and systemic health, has been identified as a sensitive indicator of the social and environmental determinants of health (9–11). In contexts of vulnerability, changes in microbial diversity and composition can reveal structural inequalities that extend beyond the clinical setting and are directly linked to living conditions.

In this study, we analyzed the diversity and composition of the oral microbiome in mothers, children, and pregnant women residing in five prisons in the State of Mexico to explore microbial variation in relation to prison-specific living conditions. We propose that the variations observed in the oral microbiome may reflect not only individual biological factors, but also social and environmental conditions associated with confinement. By providing evidence from a maternal-child perspective, this work seeks to advance understanding of the oral microbiome as an exploratory approach to studying vulnerability in contexts of confinement and to inform oral health and prevention strategies in historically marginalized populations.

## 2 Methodology

### 2.1 Study population

An observational, cross-sectional, comparative study was conducted at five Prevention and Social Rehabilitation Centers (CPRS-1–CPRS-5) in Mexico. An exhaustive sampling was carried out, resulting in a population of 43 participants as follows: 19 children raised in prison, 19 mothers of children raised in prison, in detention, and 5 pregnant women who were also in detention. All adult participants provided informed consent, and, for infants, assent was obtained and consent from their mothers. Only participants residing in prisons, who were not currently using antibiotics and had no active infections at the time of sampling, were included; those who refused to participate or whose samples had low DNA quality were excluded.

### 2.2 Sample collection

Standardized oral swabs were collected from the buccal mucosa and tongue of each participant using sterile single-use cotton swabs. Each sample was immediately placed in a sterile tube containing laboratory-prepared phosphate-buffered saline (PBS) and transported on ice under cold-chain conditions to the microbiology laboratory. DNA extraction was performed within a maximum of 5 hours after collection. All samples were handled under biosafety conditions, and negative controls were included to rule out contamination during collection and transport.

### 2.3 DNA extraction, 16S rRNA amplification, and sequencing

Total DNA was obtained from oral swabs using the DaAn Gene RNA/DNA Purification Kit (spin column) according to the manufacturer's instructions. As a pretreatment, each sample was vortexed for 1 minute and centrifuged for 1 minute to discard the supernatant and recover the initial 200 μL required by the kit. DNA concentration and purity were assessed by spectrophotometry (EPOCH, Biotek), and extracts were stored at −20°C until sequencing.

Bacterial community profiling was performed by 16S rRNA gene amplicon sequencing. The hypervariable V3–V4 regions of the 16S rRNA gene were amplified by PCR using primers 341F (5′-CCTAYGGGRBGCASCAG-3′) and 806R (5′-GGACTACNNNGGGTATCTAAT-3′). The amplicons were purified and quantified, and libraries were prepared according to standard protocols at Novogene Bioinformatics Technology Co., Ltd. (Beijing, China). Sequencing was performed on an Illumina NovaSeq 6000 using paired-end 2 × 250 bp reads.

### 2.4 Bioinformatic processing

Bioinformatic analysis was performed in R v4.4.0 (R Project for Statistical Computing, Research Resource Identifier (RRID): SCR_001905) using DADA2 v1.28 (RRID: SCR_016945). Raw reads were filtered and trimmed using the parameters truncLen = c(240, 230), maxEE = c(2, 2), and truncQ = 2. Chimeras were removed using the consensus method, and amplificon sequence variants (ASVs) were inferred from the learned error model. To standardize sequencing depth, the resulting table was rarefied to 25,000 reads per sample.

Taxonomic assignment was performed with the native Bayesian classifier of DADA2 using the SILVA 138.1 database (SILVA, RRID: SCR_006423). ASVs classified as mitochondria, chloroplasts, non-bacterial/non-archaea kingdoms, as well as those present in < 5% of samples, were removed. The refined set was imported into a phyloseq v1.44 object (RRID: SCR_013080), integrating clinical metadata, taxonomy, and count matrix for further analysis.

### 2.5 Statistical analysis

Comparative analyses were performed at two levels: between prisons (CPRS-1–CPRS-5) to identify environment-associated variations, and between groups (boys/girls and mothers/pregnant women) to assess life-stage differences.

Alpha diversity (Shannon and Inverse Simpson indices) was calculated using phyloseq (12). Statistical differences between groups were assessed via Kruskal–Wallis tests, with *p*-values adjusted using the Benjamini-Hochberg false discovery rate (FDR) correction (13) to account for multiple comparisons. Beta diversity was evaluated using weighted UniFrac distances and visualized through Principal Coordinate Analysis (PCoA). Variations in microbial community structure were tested using PERMANOVA (14) via the adonis2 function in the vegan package v2.6-6 (15) (RRID: SCR_011950) with 9,999 permutations.

Differential abundance of Amplicon Sequence Variants (ASVs) was determined with DESeq2 v1.42 (5) (RRID: SCR_000154), using the formula∼group (incorporating mothers, children, pregnant women, and prison of origin). ASVs with fewer than 20 total counts were filtered to improve power. Differential abundance was interpreted using DESeq2 effect size estimates, and ASVs were considered significantly differentially abundant if they exhibited an |log2FC| > 1 and an FDR-adjusted *p*-value < 0.01, using a conservative threshold to prioritize larger effect sizes and reduce false positives (16). All visualizations were generated using ggplot2 v3.5.1 (RRID: SCR_014601).

### 2.6 Data availability

The raw 16S rRNA gene amplicon sequencing data generated in this study were deposited in the NCBI Sequence Read Archive (SRA) under BioProject accession number PRJNA1469633.

## 3 Results

### 3.1 Sociodemographic and environmental data

Nineteen mothers residing in five CPRSs in the State of Mexico were included. Of these, six (31.5%) came from ‘CPRS-1’, two (10.5%) from ‘CPRS-2’, four (21.1%) from ‘CPRS-3’, three (15.8%) from ‘CPRS-4’, and four (21.1%) from ‘CPRS-5’. All mothers lived with at least one son or daughter within the prison environment. A total of 19 infants were included, of whom 11 were girls (57.9%), and eight were boys (42.1%). Five pregnant women were also included: one from CPRS-1, one from CPRS-4, and three from CPRS-5.

The average age of the mothers was 28.25 years (range: 19–39 years), with 27 years being the most common age. The average age of the infants was 8 months (range: 10 days–1.9 years), with a mode of 3 months. The maternal-infant areas were separated from the general population, with an average of two mothers with children per cell, and were provided with relatively adequate ventilation. General access to functional toilets and water for hygiene was reported; however, in some cases, the water had a strong chlorine odor and was not suitable for consumption, which may be a critical environmental variable. Occasional presence of urban fauna (cats and pigeons) was observed, potentially indicating environmental contamination or zoonotic risk in the centers, but without direct contact with the maternal-child areas.

### 3.2 Overall composition of the oral microbiome

**Figure 1:**
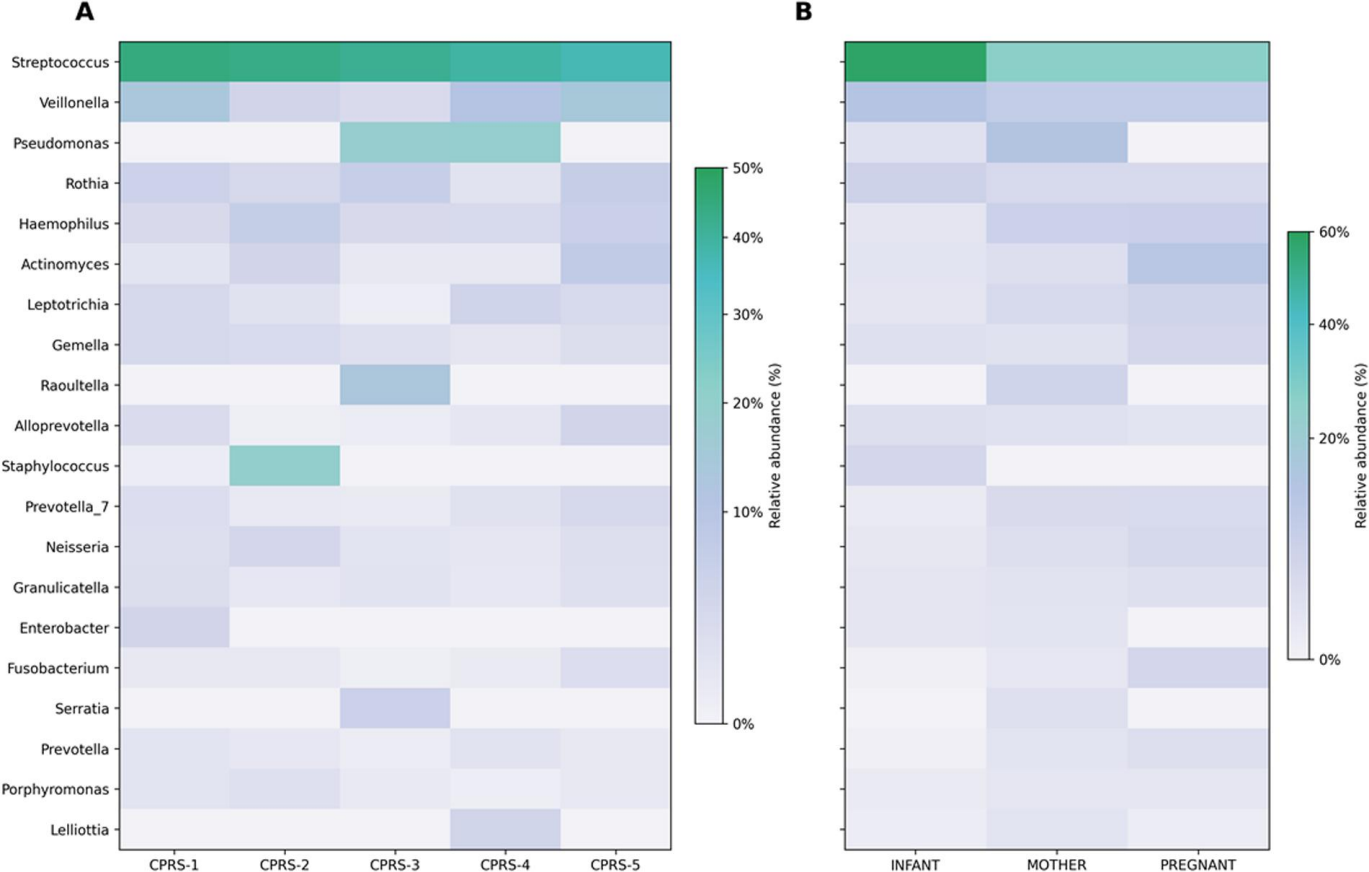
Composition of the oral microbiome in prisons and among groups of individuals. Heat maps show the relative abundance (%) of dominant oral bacterial genera based on 16S rRNA gene sequencing. **(A)** Distribution of oral bacterial genera in five prisons and social reintegration centers. **(B)** Distribution of oral bacterial genera in population groups, including children, mothers, and pregnant women. Color intensity represents relative abundance (%) and was adjusted using nonlinear normalization to improve the visualization of low-abundance taxa. Genera are shown on the *y*-axis and sample groups on the *x*-axis.

Of the 43 samples collected, 41 were successfully sequenced; two samples from the group of mothers were discarded due to poor DNA quality. Taxonomic analysis revealed a shared oral bacterial core, dominated by *Streptococcus* spp. (26%–58% relative abundance) and *Veillonella* spp. (8%–15%), present in all participants. In addition, opportunistic bacteria such as *Pseudomonas*, *Staphylococcus*, *Raoultella*, and *Enterobacter* were identified, with their distribution varying across prisons and population groups (Figure 1).

#### 3.2.1 Variation in the oral microbiome between prisons

**Figure 2:**
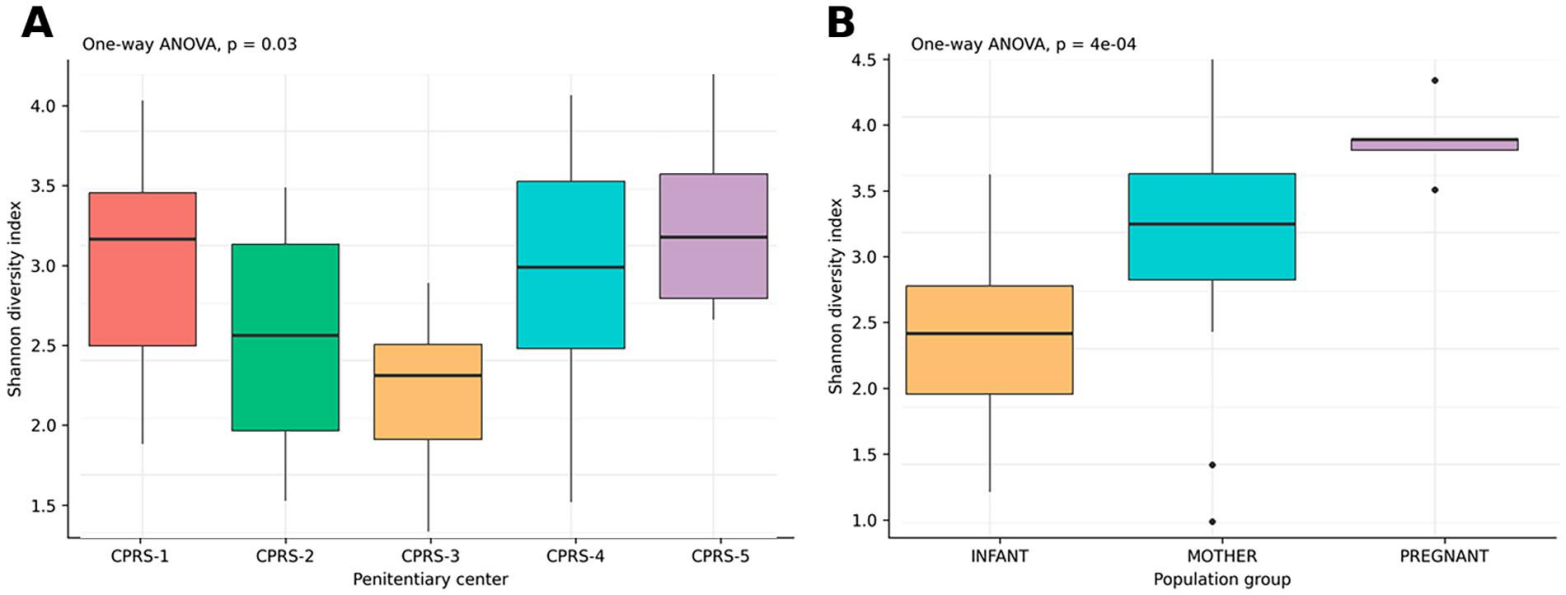
Alpha diversity of the oral microbiome in prisons and population groups. Shannon diversity index of the oral microbiome in five prisons and social reintegration centers (CPRS-1 to CPRS-5) **(A)** and in population groups, including children, mothers, and pregnant women **(B)** The boxes represent the interquartile range, the center line indicates the median, and the bars indicate 1.5 × IQR. Differences between groups were assessed using one-way analysis of variance (ANOVA).

**Figure 3:**
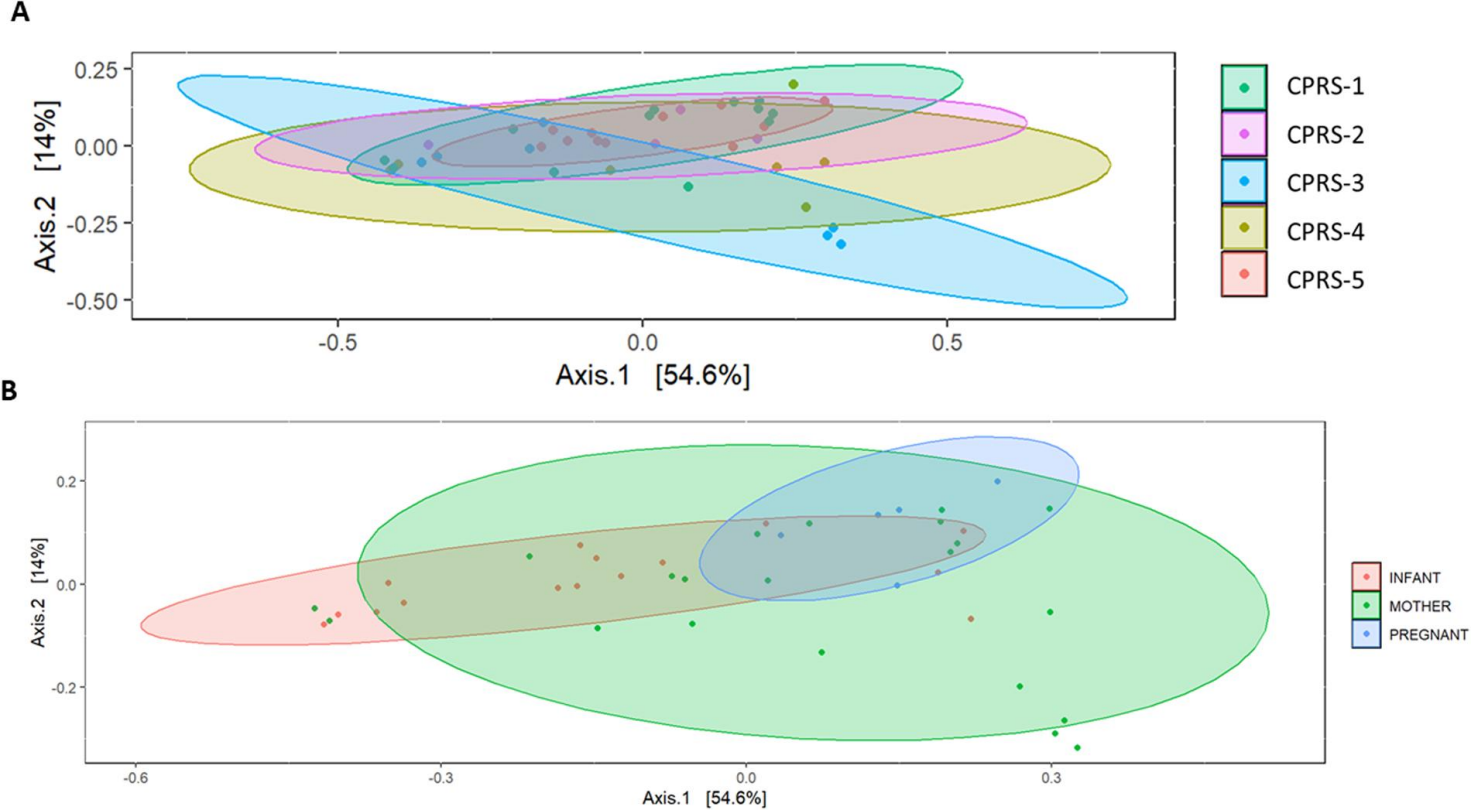
Beta diversity of the oral microbiome according to prison and population group. Principal coordinate analysis (PCoA) based on weighted UniFrac distances showing the distribution of oral microbiome samples by prison (CPRS-1 to CPRS-5) **(A)** and by population group, including children, mothers, and pregnant women **(B)** Each point represents an individual sample, and the ellipses indicate the dispersion of samples within each group.

Alpha diversity, assessed using the Shannon index, differed significantly among prisons (ANOVA, *p* = 0.03). CPRS-3 had the lowest diversity (median = 2.1; interquartile range [IQR]: 1.8–2.5), whereas CPRS-1, CPRS-4, and CPRS-5 showed higher, comparable values (medians ranging from 3.2 to 3.5). CPRS-2 showed intermediate diversity (median = 2.9) (Figure 2). Beta-diversity analysis based on weighted UniFrac distances showed partial separation among centers (PERMANOVA, R^2^ = 0.12, *p* = 0.001). CPRS-3 samples were more dispersed in the multivariate space, reflecting greater intra-center heterogeneity (Figure 3).

In terms of bacterial composition, CPRS-3 was characterized by a high relative abundance of *Pseudomonas* spp. (18.7%), while CPRS-2 showed an enrichment of *Staphylococcus* spp. (19.9%). In contrast, the other centers presented more balanced profiles, dominated by *Streptococcus* (36%–45%) and *Veillonella* (9%–12%) (Figure 1).

### 3.3 Differences according to population group

When stratified by population group, significant differences in alpha diversity were observed (Kruskal–Wallis, *p* = 0.01). Children had the lowest diversity (median Shannon = 2.2; CI: 2.0–2.4), mothers showed intermediate values and greater heterogeneity (median = 3.1; CI: 2.6–3.7), and pregnant women had the highest diversity and lowest dispersion (median = 4.0; IQR: 3.8–4.2) (Figure 2).

Beta diversity showed a clear separation between groups (PERMANOVA, R^2^ = 0.15; *p* = 0.001): the infant samples were grouped more homogeneously and differentiated from the adults; mothers exhibited high intra-group dispersion and showed partial overlap with infant samples, while pregnant women formed a compact cluster, suggesting a pregnancy-specific bacterial signature (Figure 3).

In terms of composition, children were dominated by *Streptococcus* spp. (58%) and *Veillonella* spp. (12%), while mothers had a more complex profile with *Streptococcus* spp. (26%), *Pseudomonas* spp. (13%), *Haemophilus* spp. (6.7%), *Raoultella* spp. (5.2%) and *Neisseria* spp. (4.9%). In pregnant women, in addition to the *Streptococcus–Veillonella* core, an enrichment of *Actinomyces* spp. (11.6%), *Leptotrichia* spp. (11%) and *Haemophilus* spp. (7%) was observed (Figure 1).

Differential analysis confirmed these trends: *Streptococcus* was significantly more abundant in children than in mothers and pregnant women (DESeq2, log₂FC = 1.7; FDR < 0.01), *Pseudomonas* was enriched in mothers compared to children (log₂FC = 2.1; FDR = 0.005), and both *Actinomyces* and *Leptotrichia* were significantly enriched in pregnant women (log₂FC > 2.0; FDR < 0.01).

## 4 Discusion

To our knowledge, this study is the first analysis of the oral microbiome in mothers, children, and pregnant women residing in Mexican prisons, conducted from a maternal-child perspective and within the context of social determinants of health. Our findings show that although a stable oral bacterial core composed mainly of *Streptococcus* and *Veillonella* is present, microbial diversity and composition vary across prison settings and population groups. This observation suggests that the oral microbiome may reflect biological variation associated with confinement-related living conditions, although this interpretation remains exploratory.

The identification of a microbial core dominated by *Streptococcus* and *Veillonella* is consistent with previous studies in the general population and in pediatric studies, which point to these genera as central components of the early oral microbiome (17–19). However, the high proportion of opportunistic bacteria detected in certain prisons, such as *Pseudomonas* in CPRS-3 and *Staphylococcus* in CPRS-2, suggests a microbial imbalance associated with the environmental conditions of confinement. These genera, commonly associated with hospital environments or high selective pressure, may reflect problems of hygiene, overcrowding, or deficiencies in access to water and sanitation, factors widely documented as structural determinants of health in prison settings.

From a pediatric perspective, the observed results for girls and boys are particularly relevant. The infant oral microbiome is established at birth and undergoes rapid succession in the first two years of life (20, 21). In our study, the lower alpha-diversity and marked dominance of *Streptococcus* are consistent with descriptions of early-life oral microbiome profiles (21, 22). However, the development of children within a prison environment raises important questions about how confinement-related living conditions may be associated with oral microbial patterns. Limited exposure to environmental microbial diversity, together with restrictive structural conditions, may be relevant to the early configuration of the oral community. In this sense, the infant oral microbiome may represent a useful dimension for studying oral health vulnerability in highly marginalized populations.

From a child development perspective, the presence of genera such as *Pseudomonas* and *Staphylococcus* in some prison settings may reflect environmental conditions related to sanitation and water quality. Our findings suggest that the low diversity observed in children residing in centers such as CPRS-3 may be associated with restrictive environmental conditions and potential health vulnerabilities. Therefore, interventions targeting maternal oral health and the environmental conditions of the centers may be relevant as part of pediatric preventive strategies in confined settings.

In mothers, greater interindividual heterogeneity and the presence of pathobiont or opportunistic genera ubiquitous in the environment, such as *Pseudomonas* and *Raoultella*, may reflect oral microbiome variation associated with prison-related living conditions, including water quality, hygiene, diet, stress, and limited access to dental care (23–25). Their presence in the oral cavity may be consistent with deficiencies in the hygiene chain, such as contaminated hands, poorly disinfected surfaces, or food handling under suboptimal conditions. These factors may contribute to oral dysbiosis and are relevant when considering maternal-child oral health in shared confinement environments.

A central aspect of this study is the compositional similarity observed between mothers’ and children's oral microbiomes. The partial overlap between the two groups in the beta-diversity analysis suggests shared or closely related oral microbiota, potentially associated with close cohabitation and continuous contact within the prison environment. However, this cross-sectional design does not allow us to infer mechanisms of direct transmission or temporal dynamics. From a preventive perspective, this relationship underscores the importance of considering maternal oral health in strategies aimed at improving children's oral health.

In contrast, the enrichment of genera such as *Actinomyces* and *Leptotrichia* in pregnant women in this study is consistent with previous reports describing oral microbiome variation associated with gestational hormonal changes (26). However, in contexts of social vulnerability and restricted access to dental services, this profile may be relevant for oral health monitoring during pregnancy. The presence of *Leptotrichia*, an anaerobe associated with pro-inflammatory states (27, 28) supports the importance of integrating prenatal oral health programs into prison settings.

Beyond biological differences, our results highlight the influence of the prison environment on microbial health. The lower diversity detected in CPRS-3, together with the high proportion of opportunistic organisms, illustrates how the specific environmental conditions of each facility can leave a “microbial footprint” on individuals. From a public health perspective, this suggests that the microbiome may be relevant to studying how prison-specific living conditions relate to microbial health, an innovative approach that extends beyond the traditional study of infectious diseases in prisons.

Diet, hygiene practices, and overcrowding are relevant factors in oral ecology. Diet modifies the microbial environment by altering substrate availability, whereas access to clean water and hygiene supplies may affect bacterial richness by mechanically removing biofilms and modulating daily microbial exposure (29). Overcrowding, in turn, may favor exposure to shared microorganisms and alter the immediate environmental conditions.

In this study, differences in these factors between centers could contribute to the observed variation. It is also possible that other individual and contextual factors are influencing the results. Variables such as chronic stress and mental health, which are inherent to the prison context, may alter salivary flow, pH, and secretory immunoglobulin A levels, thereby creating specific ecological niches (30). Likewise, individual clinical history, including prior antibiotic use or tobacco consumption before or during incarceration, may act as an important determinant of persistent microbial structure (31, 32). Since variables such as perceived stress level, participants’ geographic origin, and health history were not systematically measured, their potential confounding effects cannot be ruled out and should be considered in future research in confinement settings.

This study has some limitations. The sample size was small, particularly among pregnant women, and the cross-sectional design precludes the assessment of temporal changes or causal relationships. In addition, the limited sample size reduced our ability to adequately account for potential confounding factors. Variation in participants’ age (19–39 years) and duration of exposure to the prison environment (10 days–1.9 years) may have introduced substantial heterogeneity in the observed oral microbiome patterns. Other relevant variables, such as physical activity, diet, hygiene conditions, environmental exposures, and individual clinical differences, were not systematically measured and may also have influenced the results. Therefore, part of the observed variation may reflect unmeasured inter-individual heterogeneity rather than prison-related living conditions alone. In addition, the absence of a non-incarcerated comparison group limits the ability to attribute the observed oral microbiome patterns specifically to the prison context. However, sampling was exhaustive with respect to the accessible population, as it included all mothers with cohabiting children and all pregnant women deprived of liberty in prisons in the State of Mexico at the time of the study. Thus, the findings provide a robust characterization of that population in a specific temporal and contextual setting, although they are not generalizable to other populations or environments. Therefore, the results should be interpreted as exploratory and as a basis for future comparative and longitudinal research.

In conclusion, this study provides an exploratory characterization of oral microbiome patterns in mothers, children, and pregnant women residing in prisons. The identification of distinct taxonomic profiles across population groups and prison settings suggests that oral microbiome composition may be associated with both life stage and prison-related living conditions. These findings highlight the relevance of considering oral health within maternal-child care and prison health strategies. Future comparative and longitudinal studies are needed to clarify the temporal dynamics, environmental contributions, and clinical implications of these microbial patterns.

## Data Availability

The raw 16S rRNA gene amplicon sequencing data generated in this study are available in the NCBI Sequence Read Archive (SRA) under BioProject accession number PRJNA1469633.

## References

[1] Aon M Oberconz S Brasholt M. The association between health and prison overcrowding, a scoping review. BMC Public Health. (2025) 25(1):2218. 10.1186/s12889-025-23340-9 40604692 PMC12219723

[2] Bautista-Valarezo E Quirola HC Ludeña-González L Sarmiento-Andrade Y. Measuring healthcare quality in southern Ecuador prisons: a focus on health performance indicators. Disc Soc Sci Health. (2025) 5(1):20. 10.1007/s44155-025-00168-7

[3] Simpson PL Simpson M Adily A Grant L Butler T. Prison cell spatial density and infectious and communicable diseases: a systematic review. BMJ Open. (2019) 9:e026806. 10.1136/bmjopen-2018-026806 PMC6661645 31340959

[4] Sufrin C. Making mothers in jail: carceral reproduction of normative motherhood. Reprod Biomed Soc Online. (2018) 7:55–65. 10.1016/j.rbms.2018.10.018 30740546 PMC6356046

[5] Beck A Osman I Watson A Branham C Seaver B Smith A. “It’s gotta be really hard to be a mom inside right now:” a qualitative analysis on the impacts of COVID-19 on perinatal support programs for people in prison. Health Justice. (2025) 13(1):54. 10.1186/s40352-025-00359-z 40892296 PMC12403475

[6] Beaudry G Harney BL Larney S Plugge E Spaulding AC Kronfli N. Bacterial sexually transmitted infections in incarcerated populations: a systematic review and meta-analysis. Lancet Public Health. (2026) 11(1):e44–60. 10.1016/S2468-2667(25)00277-4 41500741

[7] Mundt AP Rozas-Serri E Asencio Rojas BI Morales-Rojas A Cifuentes-Gramajo PA Alvarado S. Incidence of all-cause mortality in prisons: research protocol for a global registry study and systematic literature review with meta-regression analyses. BMJ Open. (2025) 15(12):e111125. 10.1136/bmjopen-2025-111125 41407424 PMC12716586

[8] Teo AKJ Oh KH Yanagawa M Miller C Falzon D Kanchar A. Programmatic approaches to screening for tuberculosis disease: a situational analysis of seven countries in the Western Pacific region. Trop Med Health. (2025) 53(1):185. 10.1186/s41182-025-00846-x 41382194 PMC12699892

[9] Limo L Donovan J Frisbee S Gomaa N. The exposome and the human oral microbiome through the one health lens. Arch Oral Biol. (2026) 183:106504. 10.1016/j.archoralbio.2026.106504 41564716

[10] Nath S Weyrich LS Guzzo G Hedges J Tamrakar M Kapellas K. The sociobiome–oral microbiome mediates dental caries among Indigenous Australians. Front Cell Infect Microbiol. (2025) 15:1721183. 10.3389/fcimb.2025.1721183 41479542 PMC12753951

[11] Qi Q Gao C Meng X Liu W Xue Y Yan Y. Oral Microbiota dynamics across the lifespan: age, sex, race and socioeconomic influences in the US population. J Clin Periodontol. (2025) 52(11):1560–72. 10.1111/jcpe.70016 40830911

[12] McMurdie PJ Holmes S. phyloseq: an R package for reproducible interactive analysis and graphics of microbiome census data. PLoS One. (2013) 8(4):e61217. 10.1371/journal.pone.0061217 23630581 PMC3632530

[13] Benjamini Y Hochberg Y. Controlling the false discovery rate: a practical and powerful approach to multiple testing. J R Stat Soc Ser B (Stat Methodol). (1995) 57(1):289–300. 10.1111/j.2517-6161.1995.tb02031.x

[14] Anderson MJ. A new method for non-parametric multivariate analysis of variance. Austral Ecol. (2001) 26(1):32–46. 10.1111/j.1442-9993.2001.01070.pp.x

[15] Oksanen J Simpson G Blanchet FG Kindt R Legendre P Minchin P. Vegan: Community Ecology Package. Version 2.6-2. Vienna, Austria: CRAN, R Foundation for Statistical Computing (2022).

[16] Love MI Huber W Anders S. Moderated estimation of fold change and dispersion for RNA-Seq data with DESeq2. Genome Biol. (2014) 15(12):550. 10.1186/s13059-014-0550-8 25516281 PMC4302049

[17] Ahearn-Ford S Kakaroukas A Young GR Nelson A Abrahamse-Berkeveld M van Elburg RM. Spatiotemporal development of late and moderate preterm infant gut and oral microbiomes and impact of gestational age on early colonization. mSystems. (2025) 10(12):e00667–25. 10.1128/msystems.00667-25 41283679 PMC12710329

[18] Schmidt TSB Hayward MR Coelho LP Li SS Costea PI Voigt AY. Extensive transmission of microbes along the gastrointestinal tract. Elife. (2019) 8:e42693. 10.7554/eLife.42693 30747106 PMC6424576

[19] Arishi RA Cheema AS McEachran JL Gridneva Z Furst A Roman A. Development of the breastfed infant oral microbiome is associated with concentrations and intakes of human milk oligosaccharides. Nutrients. (2025) 17(22):3622. 10.3390/nu17223622 41305672 PMC12655218

[20] Kennedy B Peura S Hammar U Vicenzi S Hedman A Almqvist C. Oral microbiota development in early childhood. Sci Rep. (2019) 9(1):19025. 10.1038/s41598-019-54702-0 31836727 PMC6911045

[21] Yama K Morishima S Tsutsumi K Jo R Aita Y Inokuchi T. Oral microbiota development in the first 60 months: a longitudinal study. J Dent Res. (2024) 103(12):1249–57. 10.1177/00220345241272011 39394772 PMC11562288

[22] Kaan AM Duijster DD Ujcic-Voortman JK Haring LV Volgenant CMC Zaura E. Oral health assessment in a prospective birth cohort study. BDJ Open. (2026) 12(1):10. 10.1038/s41405-025-00395-9 41545378 PMC12811250

[23] Kotay SM Parikh HI Gweon HS Barry K Stoesser N Sarah Walker A. Biofilm removal in hospital sink drains drives unintended surges in antibiotic resistance. npj Antimicrob Resist. (2026) 4(1):5. 10.1038/s44259-025-00176-2 41593326 PMC12847690

[24] Li Y Qiu Y Gao Y Chen W Li C Dai X. Genetic and virulence characteristics of a Raoultella planticola isolate resistant to carbapenem and tigecycline. Sci Rep. (2022) 12(1):3858. 10.1038/s41598-022-07778-0 35264602 PMC8907287

[25] Appel TM Quijano-Martínez N De La Cadena E Mojica MF Villegas MV. Microbiological and clinical aspects of Raoultella spp. Front Public Health. (2021) 9:686789. 10.3389/fpubh.2021.686789 34409007 PMC8365188

[26] Kateeb E Momany E. Factors related to high dental caries experience in Palestinian pregnant women in the Jerusalem governorate: a cross-sectional study. Lancet. (2018) 391:S11. 10.1016/S0140-6736(18)30377-5 29553408

[27] Wang J Zheng J Shi W Du N Xu X Zhang Y. Dysbiosis of maternal and neonatal microbiota associated with gestational diabetes mellitus. Gut. (2018) 67(9):1614. 10.1136/gutjnl-2018-315988 29760169 PMC6109274

[28] Smid MC Dotters-Katz SK Plongla R Boggess KA. Leptotrichia buccalis: a novel cause of chorioamnionitis. Infect Dis Rep. (2015) 7(2):5801. 10.4081/idr.2015.5801 26294950 PMC4508535

[29] Santonocito S Polizzi A Isola G. “The impact of diet and nutrition on the oral microbiome”. In: Dame-Teixeira N Deng D Do T, editors. Oral Microbiome: Symbiosis, Dysbiosis and Microbiome Interventions for Maintaining Oral and Systemic Health. Cham: Springer Nature Switzerland (2025). p. 53–69. 10.1007/978-3-031-79146-8_4 40111685

[30] Nath S Zilm P Jamieson L Santiago PHR Ketagoda DHK Weyrich L. The influence of diet, saliva, and dental history on the oral microbiome in healthy, caries-free Australian adults. Sci Rep. (2025) 15(1):18755. 10.1038/s41598-025-03455-0 40436959 PMC12120111

[31] Rajasekaran JJ Krishnamurthy HK Bosco J Jayaraman V Krishna K Wang T. Oral microbiome: a review of its impact on oral and systemic health. Microorganisms. (2024) 12(9):1797. 10.3390/microorganisms12091797 39338471 PMC11434369

[32] Seo K Min JY Min KB Oh KH Ryoo SW SY S. Change of oral microbiome diversity by smoking across different age groups. Front Microbiol. (2025) 16:1714229. 10.3389/fmicb.2025.1714229 41488316 PMC12758414

